# A neuroprosthesis for control of seated balance after spinal cord injury

**DOI:** 10.1186/1743-0003-12-8

**Published:** 2015-01-21

**Authors:** Musa L Audu, Lisa M Lombardo, John R Schnellenberger, Kevin M Foglyano, Michael E Miller, Ronald J Triolo

**Affiliations:** Department of Biomedical Engineering, Case Western Reserve University, Cleveland, OH USA; Motion Study Laboratory, Louis Stokes Cleveland Department of Veterans Affairs Medical Center, Cleveland, OH USA; Department of Orthopedics, Case Western Reserve University, Cleveland, OH USA

**Keywords:** Trunk control, Seated balance, Functional Neuromuscular Stimulation (FNS), Spinal cord injury, Feedback control, Rehabilitation

## Abstract

**Background:**

A major desire of individuals with spinal cord injury (SCI) is the ability to maintain a stable trunk while in a seated position. Such stability is invaluable during many activities of daily living (ADL) such as regular work in the home and office environments, wheelchair propulsion and driving a vehicle. Functional neuromuscular stimulation (FNS) has the ability to restore function to paralyzed muscles by application of measured low-level currents to the nerves serving those muscles.

**Methods:**

A feedback control system for maintaining seated balance under external perturbations was designed and tested in individuals with thoracic and cervical level spinal cord injuries. The control system relied on a signal related to the tilt of the trunk from the vertical position (which varied between 1.0 ≡ erect posture and 0.0 ≡ most forward flexed posture) derived from a sensor fixed to the sternum to activate the user’s own hip and trunk extensor muscles via an implanted neuroprosthesis. A proportional-derivative controller modulated stimulation between trunk tilt values indicating deviation from the erect posture and maximum desired forward flexion. Tests were carried out with external perturbation forces set at 35%, 40% and 45% body-weight (BW) and maximal forward trunk tilt flexion thresholds set at 0.85, 0.75 and 0.70.

**Results:**

Preliminary tests in a case series of five subjects show that the controller could maintain trunk stability in the sagittal plane for perturbations up to 45% of body weight and for flexion thresholds as low as 0.7. The mean settling time varied across subjects from 0.5(±0.4) and 2.0 (±1.1) seconds. Mean response time of the feedback control system varied from 393(±38) ms and 536(±84) ms across the cohort.

**Conclusions:**

The results show the high potential for robust control of seated balance against nominal perturbations in individuals with spinal cord injury and indicates that trunk control with FNS is a promising intervention for individuals with SCI.

## Background

A major desire of individuals with spinal cord injury (SCI) is the ability to maintain a stable trunk while in a seated position [1]. Such stability is invaluable during many activities of daily living (ADL) such as regular work in the office or home environments, wheelchair propulsion, driving a vehicle, etc. Functional neuromuscular stimulation (FNS) has the ability to elicit contractions of the paralyzed muscles by application of measured low-level currents to the peripheral motor nerves via electrodes placed on the surface of the skin or implanted at the motor points of the various muscles of interest [2, 3]. Constant levels of pulsed stimulation are most often applied to the nerves to elicit constant force output from the muscles [4]. Such constant levels of pulsed stimulation, while suitable for maintaining static postures, are not adequate for varying postures and resisting external loads that may be acting on the trunk or other parts of the body.

Preliminary steps in designing advanced control systems for maintaining trunk posture involve studies to assess the strategy used by the intact central nervous system to mediate trunk balance in individuals with no known neurological disorders. These studies include both experimental observations of the static and dynamic behavior of trunk posture in a seated pose [5–7], as well as static and dynamic simulations with anatomically realistic musculoskeletal models of the human trunk and pelvis [8, 9]. In these studies it had been possible to explore the initial feasibility of utilizing continuous stimulation to increase trunk stiffness, vary trunk posture, and resist static perturbations. In addition to determining potential performance limitations of constant pulsed stimulation, such studies resulted in tools for evaluating more sophisticated control systems that might allow users to set their own task-dependent postures, and maintain balance even when subjected to destabilizing internal or external perturbations [9]. Perhaps the earliest work on closed-loop feedback control of the human trunk was that of Vanoncini et al. [10]. That study found that closed-loop surface stimulation of the trunk extensor muscles using a proportional, integral, and derivative (PID) controller or a linear quadratic regulator improved the stability of the trunk while in a single static posture in the presence of external disturbances, with the best outcomes obtained from purely proportional control alone.

Recent studies have shown that a self-righting control system could be deployed to automatically return the trunk to an erect posture from forward-flexed positions by using a measure of trunk tilt as a feedback signal to modulate stimulation to the trunk and hip extensor muscles appropriately [11–13]. The controller worked consistently across all five subjects with SCI in that study, notwithstanding considerable inter-subject variability in terms of injury level, voluntary and stimulated strength, and preserved sensory and motor function. The study reported in this manuscript is an extension of the controller reported in [10] whereby a feedback control system was designed to help maintain the trunk in an erect posture in the presence of external perturbations by stimulating the paralyzed muscles of the hips and trunk. We set out to explore the potential for a feedback control system using FNS to maintain an erect trunk posture when specifically measured amounts of destabilizing forces were applied to the trunk of individuals with SCI. A second objective was to determine if FNS of muscles implanted with intramuscular and epimysial electrodes could be used for effective feedback control to prevent forward falls in spite of the time delays in the force output of muscle elements. Finally, our study explored the effects of perturbation force magnitude as well as the ability to set different thresholds for controller action to kick in during forward flexion of the trunk. The volunteers relied on the backrest of the chair to support the trunk when an extension movement exceeded the erect posture.

## Methods

### Participants

Five individuals with paraplegia at different thoracic and cervical levels participated in the experiments. Table 1 shows their anthropometric and neurological characteristics. Each participant had been implanted with intramuscular and epimysial electrodes to excite hip and back muscles as listed in the last column of Table 1. All experiments were conducted by activating only the implanted muscles for each participant. As seen in Table 1 most of the participants had electrodes implanted only in the hip and back extensor muscles. One subject (S2) had an electrode implanted in a hip flexor (iliopsoas) but this was not stimulated in this study. All participants signed the consent form approved by the local institutional review board before participating in the experiments.

**Table 1:** **Summary of clinical characteristics of participants in this study**

Subject	Gender	Age	Height (cm)	Weight (kg)	Injury level	AIS Grade ^†^	Time post injury* (years)	Time post implant* (years)	Muscles activated
S1	F	42	167.6	54.4	C7	C	14.6	12.9	ES, QL, GX, SM, PA
S2	M	57	175.3	82.5	C7	B	5.3	1.5	ES, QL, IL,GM, GX, SM, PA
S3	F	58	167.6	68.6	T5-6	B	9.4	5.0	ES, GX, SM, PA, GM
S4	M	61	174.0	77.7	T6	A	14.5	7.2	ES, GX, SM, PA
S5	M	49	181.6	65.5	T10	A	6.6	1.7	ES, GX, SM, PA

Anthropometric data, injury level as well as muscles stimulated for this study are shown.

*Abbreviations*: PA, posterior portion of adductor magnus; ES, erector spinae; GX, gluteus maximus; GM, gluteus medius; QL, quadratus lumborum; SM, semimembranosus.

*At time of initial study enrollment and testing.

^†^A, motor and sensory complete; B, motor complete with sensory sparing; C, motor and sensory incomplete.

### Experimental setup

**Figure 1:**
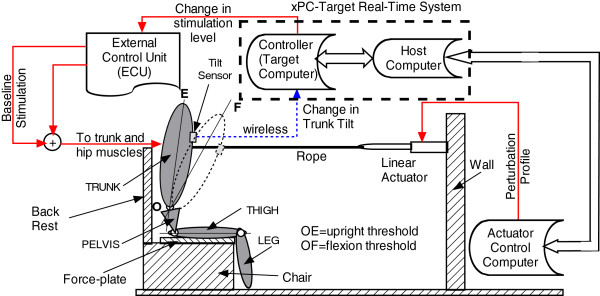
**Schematic of trunk feedback control system showing subject seated in work volume of motion capture cameras.** Three computers – actuator computer, target and host manage the real-time environment for the tests. A linear actuator applied measured pull pulses to the chest of the subject. Settings for the tilt sensor are defined as OE for the upright threshold and OF for the flexion threshold.

The volunteers sat on a chair which was placed in the work volume of a 16-camera Vicon motion capture system. A schematic of the setup is depicted in Figure 1. A wireless sensor containing a CMA3000-D01 accelerometer (VTI Technologies, Vantaa, Finland) and CC430F6137IRGC microcontroller (Texas Instruments, Dallas, TX) was employed to measure trunk tilt from the normalized component of the acceleration due to gravity in the inferior-superior direction. Thus when the subject was in an erect seated posture, the output of the sensor was highest at 1.0; while as the trunk tilted forward toward the horizontal posture the sensor output progressively reduced toward 0.0. The sensor was strapped to the sternum on the chest of the subjects below the clavicle. When switched on, the sensor streamed the x, y and z components of the acceleration wirelessly to a receiver board built into the external control unit (ECU) that generated and coordinated stimulation to the implanted muscles. In this study only the z-component was used as feedback signal since control was restricted to motion in the sagittal plane. For ease of analysis, two switching thresholds were set for the tilt of the trunk away from nominal erect posture. An upright threshold setting (OE in Figure 1) defines the tilt above which stimulation PWs to the muscles would be returned to the baseline levels, and a flexion threshold setting (OF in Figure 1) below which the stimulation PWs would be increased to the maximum levels for that subject. A program running on an xPC Target real-time control computer acquired these acceleration values and sent stimulation control commands to the ECU. xPC-Target is a dedicated computer equipped with a real-time kernel, multicore CPU, I/O and protocol interfaces. All control algorithms implemented in the Simulink software (The Mathworks, Inc., Natick, MA) were compiled in the host computer and transferred to the target computer via TCP/IP for implementation in real time.

Retro-reflective markers were placed at the left and right posterior-superior iliac spines, shoulders and on the C7 cervical spine. Others were attached to the four corners of the biomechanics force platform (Advanced Mechanical Technology, Inc., Watertown, MA) on which the subject sat. With these markers, it was possible to compute the total trunk tilt angle in the sagittal plane. This served as a confirmatory signal for the trunk tilt sensor output and also for measuring response time of the control system. A step signal from the real-time xPC Target computer synchronized the beginning and end of a trial capture with the Vicon computer.

A linear actuator (Copley Controls, Canton, MA) capable of applying forward pull forces to the trunk via a rope attached to a belt wrapped around the chest was affixed to the laboratory wall in front of the volunteer. The pull force was programmed accurately as a function of the volunteer’s body weight (BW). Depending on the ability of the volunteer to tolerate the pulls, several load levels were tested (20%, 25%, 30%, 35%, 40%, 45% and 50% of BW) starting from the lowest to the highest that could be tolerated without failure of the controller to return them to erect. Although experiments were conducted for 7 force pull levels it was observed that for most of the subjects the smaller values (20% to 30% BW) were too small to cause significant movement away from vertical while the 50% BW setting caused most of them to fall too far for the controller to act on time to restore balance. Hence subsequent details were reported only for the 35%, 40% and 45% BW magnitudes. The force pulse with respect to time was a trapezoidal shape that ramped up in 100 ms, dwelled for 100 ms and ramped down in 100 ms. The magnitude of the force output from the actuator was set in a block of the Simulink control software. The value was used by the xPC-Target computer to set the current (in mA) to the actuator amplifier.

Prior to each experiment the volunteer sat erect with their trunk supported by an adjustable back-rest of the seated platform. The purpose of the backrest was to prevent the volunteers from falling backward as the controller did not stimulate hip or trunk flexor muscles to control trunk extension.

In this study the main input variable to the muscles was the stimulation pulse-width (PW). As shown in Figure 1, the output from the accelerometer/tilt sensor was used by the Target computer to compute the changes in muscle PW to apply at the next sample time and the results communicated in real-time to the ECU for application to the users implanted muscles.

During steady state sitting, all the muscles were excited at their baseline values – being the muscle PWs suitable to keep the trunk at the erect posture. The PWs for some or all of the muscles were varied (from baseline) to oppose changes in trunk tilt as measured by the accelerometer sensor worn by the subject. Table 2 shows the baseline PW levels used for stable erect sitting as well as those used whenever the trunk tilted beyond the set threshold flexion level. For all 5 subjects, the nominal stimulation frequency was set at 20Hz (50 ms interpulse interval) for all trials. For subjects S1 and S5 however, the stimulus frequency was raised to 30Hz (33.3 ms interpulse interval) whenever the trunk angle changed beyond the flexion threshold set for that trial to recruit sufficient muscle activity to return to upright. These values were determined heuristically during the clinical profiling and calibration of the stimulation characteristics to be optimal for each subject.

**Table 2:** **Stimulation pulse-widths us for each subject**

Muscle	Side	S1		S2		S3		S4		S5	
		Baseline	High	Baseline	High	Baseline	High	Baseline	High	Baseline	High
**Erector spinae**	Right	75	100	75	34	100	150	50	250	63	250
	Left	130	250	112	30	100	250	50	130	63	250
**Quadratus lumborum**	Right	250	250	34	90	N/A	N/A	N/A	N/A	N/A	N/A
	Left	250	30	30	30	N/A	N/A	N/A	N/A	N/A	N/A
**Iliopsoas**	Right	N/A	N/A	0	0	N/A	N/A	N/A	N/A	N/A	N/A
	Left	N/A	N/A	0	0	N/A	N/A	N/A	N/A	N/A	N/A
**Gluteus maximus**	Right	200	250	62	75	60	120	250	250	100	250
	Left	40	250	75,7	112,250	60	250	250	250	80	250
**Semimembranosus**	Right	0	250	7	250	100	250	0	250	250	250
	Left	0	250	12,1	150,70	100	250	0	250	250	250
**Posterior adductors**	Right	50	250	6	250	75	250	0	200	250	250
	Left	55	200	1	250	75	225	0	250	250	250
**Gluteus medius**	Right	N/A	N/A	0	0	60	60	N/A	N/A	N/A	N/A
	Left	N/A	N/A	0	0	60	60	N/A	N/A	N/A	N/A

The ‘Baseline’ values were those used for steady state erect sitting while the ‘High’ values were those applied whenever the trunk crossed the flexion threshold. An ‘N/A’ entry implies that electrodes were not implanted to activate the muscle in that subject. Multiple entries in a cell mean that there are multiple electrodes to activate the muscle and each is controlled independently.

### Controller design

Initial testing with a proportional controller proved that the stimuli did not recruit muscle force fast enough to generate the corrective moment required to resist the perturbations. A derivative term was added to improve controller performance. It was found necessary to filter the derivative term as any noise in the sensor signal was amplified and resulted in rapidly varying muscle responses that were uncomfortable for most of the subjects. The resulting generic form of the controller equation in digitized form at time t_k_ was defined as [14]:
1

The first term in equation (1) was the proportional term while the second was the filtered derivative term. *C*(*t*_*k*_) was the controller term, D was the derivative term K_P_, and K_D_ were the proportional and derivative gains respectively and T_D_ was the derivative time constant (secs) defined as *T*_*D*_ = *K*_*D*_/*K*_*P*_; N was the derivative filter constant (2 ≤ *N* ≤ 20) and Δt was the sampling time (secs). A normalized form of the error signal e was computed using the equation:
2

In (2), a was the current accelerometer reading, *a*_min_ was the upright threshold setting (OE in Figure 1), and *a*_max_ the flexion threshold setting (OF in Figure 1). The ability to vary the upright and flexion thresholds in equation (2) would test the potential for the user to set the limits of the control of the trunk depending on the specific task at hand – if the task would require the trunk to bend more forward, then the flexion threshold could be set to a lower value than if the task required the trunk to remain closer to erect.

For FNS application, the output from the controller as defined by equation (1) passed onto the system actuators (the muscle elements) whose dynamics was modeled by a linear recruitment curve that modulated force between baseline PW and saturation PW as in the following equation:
3

In equation (3),  was the PW setting for the ith muscle,  was the baseline PW applied to the ith muscle whenever the trunk tilt was between erect and *a*_min_;  was the saturation PW applied to the ith muscle whenever the trunk tilt exceeded the set flexion threshold of *a*_max_. The baseline PWs were earlier determined as the optimum settings that allow for a stable erect seated posture. The saturation PWs were those found to be the maximum above which no additional muscle force could be elicited from the muscle.

**Figure 2:**
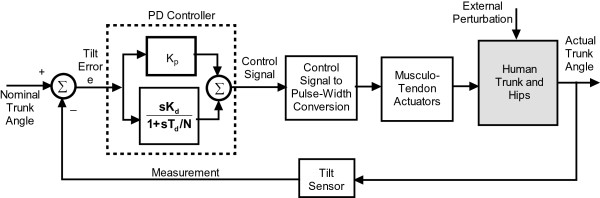
**Flow diagram for the feedback control of erect seated posture.** The error in trunk tilt measured a body-mounted sensor provides input to a PD controller which produces a normalized control signal that is converted to PW values to be applied to all the extensor muscles of the hip and trunk. s is the Laplace transform parameter.

The controller flow diagram is shown in Figure 2. The Figure shows the relationship between the control signals and the system actuator sub-system; which in this study consisted of the implanted muscle elements.

For each subject 18 trials were analyzed. The combination of three perturbation amplitudes (35%, 40% and 45% BW) and 3 flexion threshold levels (0.85, 0.75 and 0.70) led to 9 conditions. An open source random allocation software [15] was used to generate a random order for all trial conditions. After the first 9 random combinations were executed, the process was repeated again for the same 9 combinations. Each trial consisted of the application of three pulses (at the same pull magnitude and flexion threshold) that were programmed to be applied at 10s intervals to allow the volunteer adjust to the nominal seated position before the next pull. In each trial, the upright threshold was fixed at around 0.95 which was close to the assumed erect value of 1.0.

### Figures of merit

Subjects were unable to tolerate the force pulse perturbations without the controller active. The applied disturbances, even at the lower level of 35%BW, resulted in losses of balance and rapid forward flexion of the trunk that risked potentially injurious falls from the chair. For safety reasons, it was therefore impossible to compare the controller to a baseline consisting of stimulation suitable for quiet sitting.

Two global performance metrics were calculated to quantify the operational effectiveness of the control system for each subject: a) settling time and b) response time. Both can be deduced from the data in the experiments in this study.

- Settling Time

The settling time was defined as the time elapsed from the application of an ideal instantaneous step input to the time at which the output (trunk angle) has returned and remained within a specified set band. In this study, we chose to define it as the time from the application of the pulse perturbation input to the time the trunk angle returned and remained within 5% of its original steady-state value prior to the perturbation.

b) Response Time

The response time was defined as the time elapsed from the onset of the stimulus change from the controller to the time when the trunk angle reached 10% of its peak amplitude following the perturbation.

## Results

**Figure 3:**
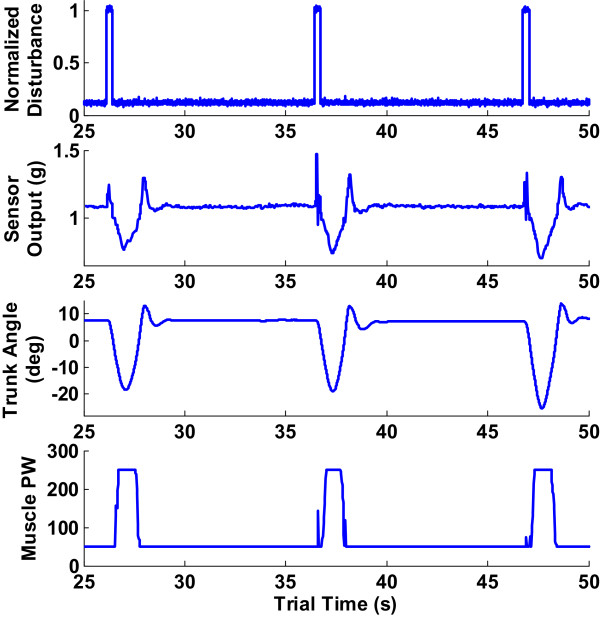
**Typical complete trial consisting of 3 cycles of perturbation force application from a trial with Subject S1.** Top plot is the normalized disturbance, second plot is the accelerometer sensor output (in g’s) which was used as a measure of trunk forward flexion, third plot is the trunk angle computed from marker data and last plot is the muscle pulse-width for the erector spinae muscle activated as a consequence of change in trunk tilt.

The results reported here were the averaged values over 6 cycles (2 trials for each of the 9 conditions with 3 test cycles per trial). Figure 3 shows the typical raw data of all three test cycles in one trial for Subject S1. The top plot in this figure was the disturbance signal which had been normalized from the actual current value in mA returned from the actuator amplifier. First the subject was allowed to adjust until a steady erect posture was achieved. This took between 10 to 25 seconds. Thereafter the linear actuator applied 3 pulls at 10 second intervals, followed by another period of return to nominal erect posture before the trial ended. The small overcorrection noted in the trunk angles (third trace from the top) was an indication that the applied stimulation was slightly larger than necessary to return to erect exactly and the subsequent forward return toward erect was due to rebound as the trunk hit the backrest of the chair.

### Effect of trunk tilt on pulse-width

**Figure 4:**
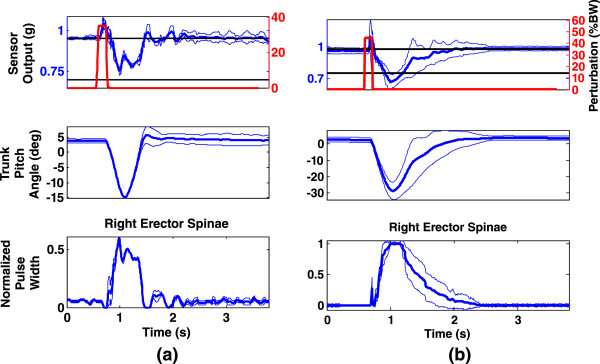
**Typical result of feedback perturbation rejection control experiment with Subject S4. (a)** Pull magnitude = 35%BW; flexion threshold = 0.70 where saturation pulse-width was not attained and **(b)** Pull magnitude = 45%BW; flexion threshold = 0.75 where saturation pulse-width was attained. The thick lines in the plots represent response means over 6 pull cycles ±1 standard deviation (thin lines). The trunk angles in the middle subplots were calculated using the marker positions captured with the Vicon cameras.

Figures 4(a) and (b) show the results of perturbation with the disturbance profile (red) and resultant sensor output (blue) at the top two sub-plots for subject S4. Figure 4(a) shows the case when the input was not large enough to cause the trunk to tilt far enough to elicit saturation of PWs while 4(b) depicts the case when the disturbance was large enough to cause PW saturation (‘High’ values in Table 2). The middle sub-plots show the trunk angle computed from the coordinates of the reflective markers as captured by the motion capture cameras. The lower subplots depict changes in the muscle PWs as specified by the controller. The PW plots were normalized so that zero normalized PW represents baseline stimulation while normalized PW equal to 1 implied saturation (High) level PW (see Table 2). From these plots it is clear that the PW changes occurred in accordance with the changes in trunk tilt or sensor output. Whenever the sensor output exceeded the set maximum flexion threshold, the PW values reach and remain at the saturation levels as evidenced by the flat portions of the PW plots at the bottom of Figure 4(b). It should be noted that the plots for perturbation pulse, sensor output and muscle PWs were all the real-time values as measured within the xPC-Target control computer. The only variable external to these was the trunk angle measured by the Vicon motion capture cameras (middle sub-plots in Figure 4).

### Effect of perturbation amplitude

**Figure 5:**
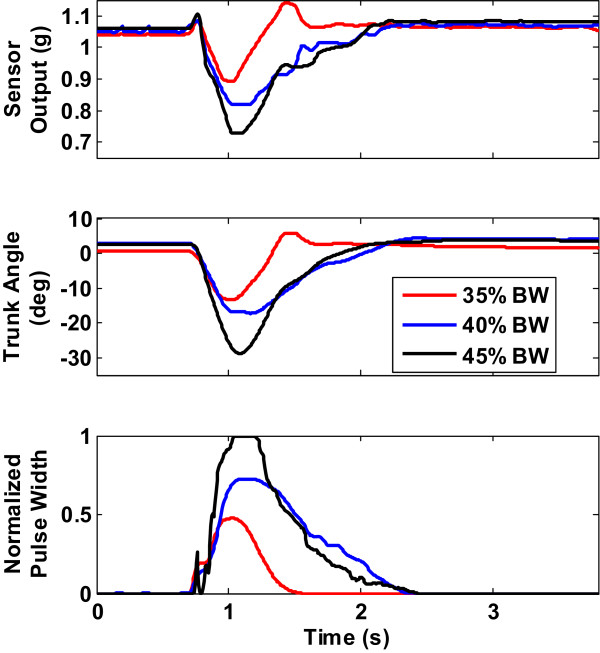
**Typical response showing sensor output, trunk angle and muscle stimulus for different pull amplitudes for Subject S4.** In all cases, the flexion threshold was set at 0.75. With lower pull magnitudes the trunk did not reach the set flexion threshold before muscle action restored it to erect. With the larger pull magnitude of 45%BW, the trunk flexed up to the set flexion threshold before muscle action was strong enough to restore it to erect.

Figure 5 shows a plot of the average sensor output, trunk angle and normalized PW as functions of perturbation magnitude for Subject S4 with the flexion threshold fixed at 0.75. From this figure, it is apparent that the smaller the perturbation magnitude the smaller the response (sensor output and trunk angle) and also the smaller the settling time. The peak differences for the three pull amplitudes were of the order of 1.0:1.5:2.3 for sensor output, about the same ratio for trunk angle and of the order of 1.0:1.5:2.1 for normalized PW magnitudes. These ratios imply that setting a flexion threshold did not mean that the trunk tilt must reach it before muscle action was large enough to return the trunk to erect. If the disturbance was not large enough restoration to erect would occur before the trunk reached the set flexion threshold. Table 3 shows the peak response ratios for all 3 pull magnitudes and for all 5 subjects. From the first 11 rows of this table it appears that the ratios for the other pull magnitudes and flexion thresholds are of the same order of magnitude for all subjects.

**Table 3:** **Ratios of the peaks of the responses (trunk tilt, trunk angle and muscle pulse-width) as functions of changes in the pull magnitude (35%BW:40%BW:45%BW) and changes in the flexion thresholds (0.85:0.75:0.70) for all 5 subjects**

	Variable		Subject				
		Condition	S1	S2	S3	S4	S5
**Ratios of response to varying pull magnitudes: 35%BW:40%BW:45%BW**	**Tilt**	0.70	1:1.2:2.2	1:1.4:2.2	1:1.2:2.5	1:1.4:2.4	1:1.3:2.3
		0.75	1:1.6:2.8	1:1.6:2.3	1:1.3:2.1	1:1.5:2.3	1:1.6:2.3
		0.85	1:1.4:2.1	1:1.5:2.0	1:1.3:2.8	1:1.4:2.2	1:1.5:2.9
	**Angle**	0.70	1:1.2:2.7	1:1.3:2.6	1:1.2:2.4	1:1.3:2.5	1:1.3:2.4
		0.75	1:1.5:2.0	1:1.5:2.3	1:1.6:3.0	1:1.5:2.3	1:1.5:2.0
		0.85	1:1.2:2.3	1:1.3:2.1	1:1.3:2.3	1:1.3:2.9	1:1.3:2.5
	**PW**	0.70	1:1.4:2.3	1:1.2:2.2	1:1.4:2.7	1:1.4:2.5	1:1.4:2.7
		0.75	1:1.7:2.2	1:1.5:2.1	1:1.4:2.5	1:1.5:2.2	1:1.9:2.3
		0.85	1:1.6:2.2	1:1.4:2.1	1:1.8:2.2	1:1.5:2.4	1:1.8:2.9
**Ratios of response to varying flexion thresholds: 0.85:0.75:0.70**	**Tilt**	35%BW	1:1.3:2.3	1:1.6:2.1	1:1.4:2.7	1:1.5:2.2	1:1.4:2.3
		40%BW	1:1.6:2.1	1:1.3:2.1	1:1.5:2.0	1:1.5:1.9	1:1.6:2.2
		45%BW	1:1.5:1.7	1:1.3:2.5	1:1.4:2.8	1:1.6:2.4	1:1.4:2.2
	**Angle**	35%BW	1:1.4:2.4	1:1.5:2.4	1:1.4:2.0	1:1.3:2.1	1:1.6:2.6
		40%BW	1:1.4:2.9	1:1.3:2.3	1:1.5:2.4	1:1.5:2.8	1:1.4:2.2
		45%BW	1:1.9:2.5	1:1.8:2.3	1:1.5:2.2	1:1.5:2.8	1:1.3:2.5
	**PW**	35%BW	1:1.7:2.3	1:2.0:2.2	1:1.7:2.1	1:1.7:2.4	1:1.8:2.3
		40%BW	1:1.4:2:7	1:1.4:2:3	1:1.4:2:6	1:1.2:2:8	1:1.3:2.5
		45%BW	1:1.0:1.0	1:1.0:1.0	1:1.0:1.0	1:1.0:1.0	1:1.0:1.0

### Effect of flexion threshold settings

**Figure 6:**
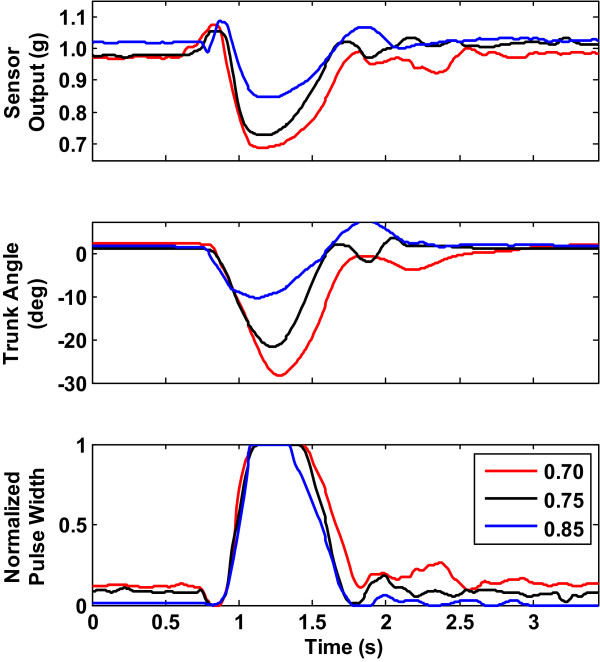
**Typical response trunk tilt, angle and muscle stimulus for different flexion threshold settings at the same pull magnitude of 45% BW for Subject S1.**

Figure 6 shows the effect of varying the flexion threshold on sensor output, trunk angle and PW for Subject S1. The plots were selected for the same pull magnitude of 45% BW while the flexion thresholds were the values set at 0.85, 0.75 and 0.70. Although we also tested at lower values of flexion threshold (0.65 and 0.5), these were not reported because the controller was unable to restore balance to most of the subjects (3 out of 5) to erect at these lower settings. The results indicate that with large disturbance the trunk would tilt far enough to reach the set flexion threshold before returning to erect. The ratios of the peaks for the three flexion threshold values were of the order of 1.0:1.5:1.7 for sensor output, 1.0:1.9:2.5 for trunk angle and 1.0:1.0:1.0 for PW. The ratios of the peaks of the sensor output, trunk angle and PW indicate that the controller restored the trunk to erect whenever the disturbance was not too large (up to around 40% BW) even though for larger disturbances around 45% BW the PW’s reached their saturation levels. The ratios of the peaks of the trunk tilt, trunk angle and PW for other values of the flexion threshold are depicted in the last 9 rows of Table 3 for all 5 subjects. From this table, the ratios have similar orders of magnitude for the different conditions in all the 5 subjects. Generally for both perturbation amplitude and flexion threshold settings, the ratios tend to increase with increase in disturbance magnitude for various thresholds. They also tend to increase with increasing threshold for a given disturbance magnitude until PWs are maxed out across all subjects in which case the ratios become close to 1:1:1 as the last line of Table 3 shows.

### Settling time

**Figure 7:**
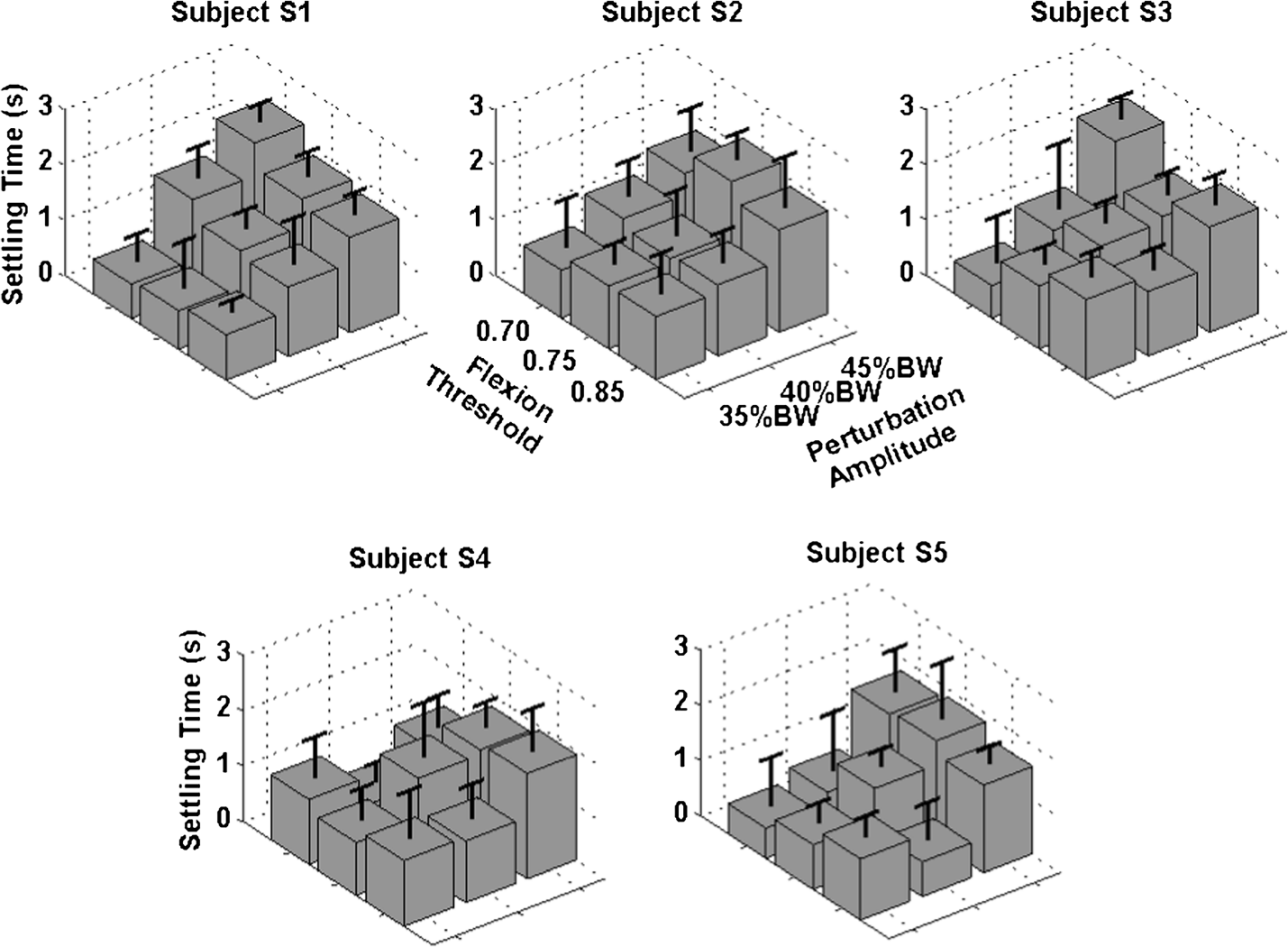
**Mean settling times for trunk angle for each of the 5 subjects shown by the grey blocks.** The leftward axis was the flexion threshold, the rightward the perturbation amplitude, while the vertical axis was the settling time in seconds. The thick black lines are the error bars that represent the standard deviations from the means.

The mean settling times for the 5 subjects are shown in the bar-graphs in Figure 7 for all the 9 conditions. The mean values varied between 0.5 and 2 s even though the variations over these mean values were rather large. Two of the subjects (S1 and S2) displayed smaller variations in settling time between conditions while the other three show much wider variations between conditions as evidenced by the lengths of the error bars. For most of the subjects, the tendency was for the mean settling times to be larger (of the order of 2 secs) as the perturbation magnitude increased. For some of the subjects (S1, S4 and S5), however, such larger mean settling times also occurred at the 40% BW setting. There did not seem to be any similar pattern for variations in the flexion threshold.

### Response time

**Figure 8:**
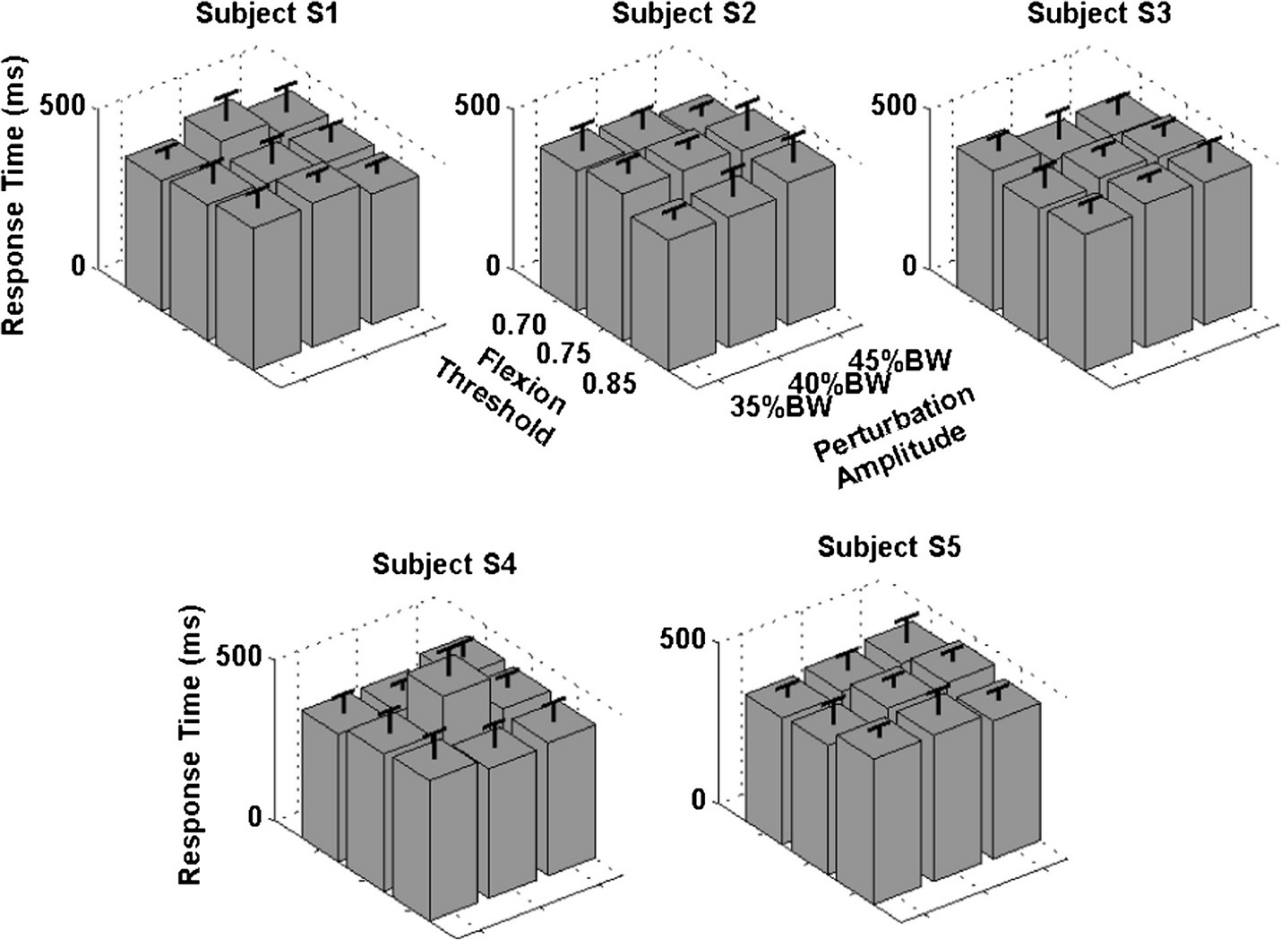
**Means of the response times for trunk angle for each of the 5 subjects shown by the grey blocks.** The leftward axis was the flexion threshold, the rightward the perturbation amplitude, while the vertical axis was the response time in milliseconds. The thick black lines are the error bars that represent the standard deviations from the means.

The mean response times for the 5 subjects are shown in the bar-graph in Figure 8 for all the 9 conditions. Typically the system response time mean values were of the order of 400 ms for all subjects with lesser variations around the mean values than the settling times. Also, there appeared to be small variation in response time between conditions for all subjects.

## Discussion

We have developed a feedback control system for control of seated balance in the sagittal plane after spinal cord injury and tested the control system in 5 individuals: 3 with thoracic and 2 with cervical level injuries. The feedback control system used the component of acceleration due to gravity to measure the overall tilt of the trunk. The control system worked as expected to restore seated balance in all 5 subjects tested with perturbation amplitudes up to 45% BW and with flexion thresholds as low as 0.7 as illustrated in Figures 7 and 8 and in Table 3. With larger perturbation amplitudes and thresholds lower than these values, the controller failed in most cases to restore the trunk to the erect. Inherent in the control equation (3) was the assumption that each of the muscle elements contributed equivalently to the control of the trunk tilt. This ignored the potential that in the intact system muscle elements may be recruited in certain order depending on the level of control requirements. In addition, this control architecture assumed all time delays in the production of force by the muscles were negligible. Finally, the force recruitment of the muscle elements was assumed to be linear between the baseline and saturation (high) PWs.

In the work reported in [8–10], a similar feedback control system was described in which the feedback signal was based on the same accelerometer sensor used in the current study. The previous studies differed in three main ways from the current study. First, the change in trunk angle was initiated by the subjects by letting go of the trunk support with their upper extremities unlike in the current study where the change was initiated by applying a measured pulse perturbation; secondly, the change in PW above the baseline level was applied only after the trunk had crossed the flexion threshold whereas in the current study the change in stimulation from baseline occurred at all times that the trunk angle deviated from the set value for erect posture. Finally, the PWs applied were always the maximum (high) level in order to restore the trunk to erect. The stimulation would remain at that value until the trunk crossed a lower (upright) threshold level when pulse-widths were restored to their baseline levels.

Another recent attempt to use feedback control for seated balance was that reported by Vanoncini et al. [7]. Our approach differed from that reported in [7] in three principle ways. First, whereas the system in [7] was restricted to control of the thorax alone (with the pelvis held fixed), our study examined the potential for control of the whole trunk with movement about both the lumbar and hip joints. We believed this would be the situation when the subjects were to use the system in a home or office environment. Secondly, the study in [7] examined the system with muscle outputs from electrodes placed on the surface of the skin. All our subjects had implanted electrodes to the hip and back muscles and had been using their systems for other activities such as exercise, standing and walking. Since implanted systems are known to be more robust in terms of their repeatability, accessibility and selectivity [16], this may explain our ability to control not only the lumbar joints but also the hip joints at the same time. Finally, our approach included the application of baseline muscle excitations at the erect seated posture. That stimulation level was an important prerequisite for stable control since it provided the effort needed to maintain the erect seated posture without fully relying on the backrest of the instrumented chair. In our subjects it was impossible to achieve that posture with no baseline stimulation.

Whereas in [7] best results were obtained using pure proportional gain alone, we got adequate control with a proportional derivative control element. Future work will explore the potential impact of using all control terms (proportional, derivative and integral) or even more advanced control systems such as model predictive control, fuzzy control, etc.

Vanoncini et al. [7] set a maximum value of 3 s for the settling time, although the return threshold limit was set at ±1° and the maximum oscillation about the reference trunk angle was set at ±5°. Considering that we allowed movement up to 30° of flexion from the nominal posture, we set the threshold at ±5° around the value prior to perturbation application. With these specifications, the system was able to recover the trunk to erect in most subjects in less than 3 s (average settling time for each of the subjects shown in Figure 7); a feat which was never achieved in the experiments in [7] where the minimum settling time was around 5 s. This difference could be attributable to the larger number of muscles utilized in the current study (see Table 1) as well as to the use of implanted electrodes as opposed to the surface electrodes on erector spinae muscles alone used in [7].

The closest concept to system response time was that of electromechanical delay (EMD) [17, 18]. The EMD was generally defined as the delay between the onset of muscle electrical activity (as measured by EMG) and measurable tension output from the muscle. This value has been estimated to lie between 30 and 100 ms [17] for intact human muscle. Values of EMD ranging between 14 to 105 ms were measured as the delay between onset of application of surface stimulation to isometric force output of quadriceps muscles in able-bodied individuals [19]. In the current study, the system response time was around 450 ms. The large difference between this value and typical EMD values could be attributable to the additional delay between onset of muscle force output and actual changes in limb movement; a phenomenon that could be attributable to the inertia in the system. Overall, these large delay values further underscore the need for close attention of time delays as important factors in the design of control systems for balance studies with FNS after SCI [20, 21].

Even though the results reported here indicated encouraging outcomes for control of seated balance after SCI, there were a number of limitations that restrict the applicability of the results in a way. First, the muscle recruitment curves were assumed to be linear. In general most muscle elements exhibit nonlinear force-PW characteristics [22]. The effect of including these nonlinearities may lead to more refined results; but we believe that in control of motions where the trunk has moved too far away from nominal erect posture it is not clear if the impact would be significant. Second, only motion in the sagittal plane was studied. More interesting control would involve motion of the trunk in the whole workspace in front and to the sides of a typical seated individual. Thirdly, in this study all the muscle elements were assumed to contribute equivalently to the restoration moment applied at the lumbar and hip joints. No attempt was made to distribute the restoration moment in other ways that may be more in line with what the intact central nervous system may be doing. Such a distribution may require the use of optimization algorithms. A smoother control of perturbation rejection may be achievable with such control systems although the computational burden may be too high for real-time control applications. Fourth, the control system studied here can be considered unidirectional (flexion) since it mainly acted to prevent trunk flexion. Extension movements that went beyond erect were arrested by the backrest of the chair. Similar to [7], there was no attempt to control any extension movements with muscle stimulation. Such control in the anterior as well as posterior directions would require a robust set of hip or trunk flexor muscles in addition to the trunk extensor muscles. The most prominent ones that could be recruited are rectus abdominis, rectus femoris or iliopsoas. The effect of such bidirectional trunk control is the subject of future studies and beyond the scope of this project. Also, in applications where the user is subjected to sustained activation of the hip and trunk muscles to maintain a static posture, such as possible during wheelchair propulsion or driving a vehicle, muscle fatigue could easily compromise the effectiveness of any control system designed to make dynamic corrections to postural disturbances. The impact of fatigue will be an important and interesting topic to explore and a challenging issue to deal with in future designs of control systems for seated balance. Finally, the control system reported in this paper describes the ability to control seated posture in the presence of external impulsive perturbations. It was difficult to replicate the experiments with no stimulation above baseline levels as these could result in potential injury to the subjects. Asking the subjects to use their upper arms or using protective harnesses could all confound the results and make it impossible to make sensible comparisons with the cases where stimulation was applied. Control systems for cases where the trunk was unperturbed by large external force pulses and allowed to fully flex forward freely under the influence of only gravity have been reported in [9, 10]. In these previous studies, comparisons were possible for cases with and without stimulation, while in the present study the application of perturbing force pulses without stimulation beyond baseline was considered to be too dangerous to pursue at this time.

## Conclusions

These initial results support the feasibility of automatically controlling seated balance with FNS. Perturbations up to 45% BW were successfully rejected by the controller. This implied that such a controller would be suitable for maintaining a stable trunk during activities of daily living and prevent falls during wheelchair propulsion or when driving a vehicle without the need for restraining devices that would otherwise decrease the workspace around the user. Further studies should include control in the coronal plane as well and also ability to set and attain any desired posture other than the erect posture within the typical work volume of a seated operator.

## Abbreviations

SCI: Spinal cord injury
FNS: Functional neuromuscular stimulation
BW: Body weight
ADL: Activities of daily living
ECU: External control unit
PID: Proportional, integral and derivative.
